# Cystic Echinococcosis Epidemiology in Spain Based on Hospitalization Records, 1997-2012

**DOI:** 10.1371/journal.pntd.0004942

**Published:** 2016-08-22

**Authors:** Zaida Herrador, Mar Siles-Lucas, Pilar Aparicio, Rogelio Lopez-Velez, Alin Gherasim, Teresa Garate, Agustín Benito

**Affiliations:** 1 National Centre for Tropical Medicine, Health Institute Carlos III (ISCIII in Spanish), Madrid, Spain; 2 Network Biomedical Research on Tropical Diseases (RICET in Spanish), Madrid, Spain; 3 Laboratory of Parasitology, Instituto de Recursos Naturales y Agrobiología de Salamanca (IRNASA), Consejo Superior de Investigaciones Científicas (CSIC), Salamanca, Spain; 4 National Referral Centre for Tropical Diseases, Infectious Diseases Department, Ramón y Cajal Hospital, Instituto Ramón y Cajal de Investigación Sanitaria, Madrid, Spain; 5 National Centre of Epidemiology, Health Institute Carlos III (ISCIII), Madrid, Spain; 6 National Centre of Microbiology, Health Institute Carlos III (ISCIII), Madrid, Spain; University of Zurich, SWITZERLAND

## Abstract

**Background:**

Cystic echinococcosis (CE) is a parasitic disease caused by the tapeworm *Echinococcus granulosus*. Although present throughout Europe, deficiencies in the official reporting of CE result in under-reporting and misreporting of this disease, which in turn is reflected in the wrong opinion that CE is not an important health problem. By using an alternative data source, this study aimed at describing the clinical and temporal-spatial characteristics of CE hospitalizations in Spain between 1997 and 2012.

**Methodology/Principal Findings:**

We performed a retrospective descriptive study using the Hospitalization Minimum Data Set (CMBD in Spanish). All CMBD’s hospital discharges with echinococcosis diagnosis placed in first diagnostic position were reviewed. Hospitalization rates were computed and clinical characteristics were described. Spatial and temporal distribution of hospital discharges was also assessed. Between 1997 and 2012, 14,010 hospitalizations with diagnosis of CE were recorded, 55% were men and 67% were aged over 45 years. Pediatric hospitalizations occurred during the whole study period. The 95.2% were discharged at home, and only 1.7% were exitus. The average cost was 8,439.11 €. The hospitalization rate per 100,000 per year showed a decreasing trend during the study period. All the autonomous communities registered discharges, even those considered as non-endemic. Maximum rates were reached by Extremadura, Castilla-Leon and Aragon. Comparison of the CMBD data and the official Compulsory Notifiable Diseases (CND) reports from 2005 to 2012 showed that official data were lower than registered hospitalization discharges.

**Conclusions:**

Hospitalizations distribution was uneven by year and autonomous region. Although CE hospitalization rates have decreased considerably due to the success of control programs, it remains a public health problem due to its severity and economic impact. Therefore, it would be desirable to improve its oversight and surveillance, since officially reported data are underestimating the real burden of CE in Spain.

## Author Summary

Cystic echinococcosis (CE) remains an important health problem in many regions of the world, including those where control programs have been implemented. In Spain, available information on CE is incomplete, and its importance tends to be underestimated due to underreporting and to the lack of compulsory notification. In this paper we used hospitalized CE discharge data to describe CE main epidemiological characteristics and CE geographical and temporal trends in Spain. Although CE hospitalization rates have decreased considerably in the last years, the overall economic losses estimations due to CE were still rather high in Spain. The need for a continuous and well-defined source, with national coverage, of epidemiological data on CE is emphasized in this paper, not only to have a more accurate picture but to monitor the control activities in place.

## Introduction

Human echinococcosis (HE) is a zoonotic disease caused by the larval cystic stages of cestode species in the genus *Echinococcus*. The infection, one of the 17 neglected tropical diseases listed by the World Health Organization (WHO), has a cosmopolitan distribution and is maintained through a variety of domestic, synanthropic, and sylvatic cycles [1]. The two most important species are *E*. *granulosus* (*sensu strictu*), the cause of cystic echinococcosis (CE), and *E*. *multilocularis*, the cause of alveolar echinococcosis (AE) [2]. HE is acquired by ingesting eggs, coming from the faeces of definitive hosts (dogs, wolves and other canids), that harbour the adult *Echinococcus* worms in their small intestine. Humans can be exposed to these eggs by "hand-to-mouth" or “food-to-mouth” transfer or contamination.

CE is the cause of significant losses in endemic areas [3]. It is found in Africa, Europe, Asia, the Middle East, and Central and South America. The parasites causing cystic echinococcosis (CE) are transmitted to man and domestic animals either directly or indirectly from dogs. The highest prevalence rate is found in populations that raise sheep. Although not yet been well-defined, some reported risk factors for CE human infection include uncontrolled dogs living closely with people, uncontrolled slaughter of livestock, and unsanitary living conditions [4]. Population at high risk of CE include shepherds, butchers, slaughters, veterinarians, and all whose job requires them to work closely with animals at higher risk of the infection [5,6].

Humans may acquire CE infection from wild or domestic animal hosts, but the parasite cannot be directly transmitted between humans. Therefore from a control viewpoint, treatment of CE cases will have no effect on pathogen transmission [7]. Where the importance of CE has been recognized, control programs have been implemented, leading to a drastic reduction in its prevalence [8]. However, the decrease of CE cases has also caused the discontinuation of control measures and the exclusion of this disease from the list of notifiable diseases [9].

In Europe, *E*. *granulosus* has an uneven geographical distribution with very low prevalence in some of the northern and central European countries, with medium endemicity in others, while highly endemic in the Mediterranean and Some Eastern countries [10]. In Spain, CE is one of the most important anthropozoonoses in terms of incidence and morbidity [9]. In 1985, there were about 1,000 new human cases (about 2.5/100,000 per year). Since the eighties of the last century, a continuous decrease of CE has been reported, i.e. there were about 600–700 new cases per year in the 1980s and 500-300/year in the 1990s. In 1997, the incidence rate dropped to 0.78/100,000per year. These excellent results were achieved after the implementation of national control programs by the autonomous communities (CC.AA in Spanish), mainly based on slaughterhouse hygiene, public education and the use of periodic treatment of dogs with praziquantel [10].

The CE notification in Spain was mandatory at national level from 1981 to 1996 [11]. Since 1996, the regional autonomous authorities of each CC.AA became fully in charge of the financing and implementation of the control programs. From 1997 on, CE disease is only surveyed by those autonomous communities considered endemic [13]. Currently, the national epidemiological surveillance network is based on 3 interdependent systems: Compulsory Notifiable Diseases (CND), Outbreak Alerts (OA) and Microbiological Information (MI). The CND is considered the universal notification system, providing the official figures published in the Spanish Report on Trends and Sources of Zoonoses by the European Commission [14]. The performance and coverage of these systems in CE reporting is very limited both quantitatively and qualitatively, due to a number of factors derived from their operating designs and the particular biological features of CE [13,15]. Other possible source of information is the Centralized Hospital Discharge Database (CMBD in Spanish). The CMBD has been recently used to estimate the incidence of CE in the province of Salamanca [11] and in the autonomous region of Madrid [16]. Both surveys found relevant epidemiological data showing higher incidence rates than those reported by the CND. This study aims to describe the hospital admissions and patients’ characteristics related to CE disease at a national level to get a better picture of the current epidemiological scenario after the implementation of regional control programs.

## Methods

### Data analysis

We performed a retrospective descriptive study using CMBD information on CE related hospitalizations in Spain between January 1st, 1997 and December 31st, 2012. CMBD database receives notification from around 98% of the public hospitals in Spain [17]. The National Health System (NHS) provides free medical care to 99.5% of the Spanish population, although those persons not covered by the NHS can be attended at the public hospitals. Private hospitals represent only a small proportion of all hospital admissions. Since 2005, CMBD also has a gradual coverage from private hospitals [18].

All CMBD’s hospital discharges with any type of CE diagnosis placed in first diagnostic position were reviewed. The first diagnostic position is the main cause of hospitalization. International Classification of Diseases, Ninth Revision, Clinical Modification (ICD 9 CM), the ICD version employed during the study period, was used for this purpose (Table 1) [19]. Those hospital discharges with first diagnosis of *E*. *multilocularis* infections (ICD 9 codes: 122.5–122.7) were excluded from the analysis.

**Table 1:** Main diagnosis in human echinococcosis hospitalizations, 1997–2012, Spain.

ICD-9-CM code	Type (according to ICD-9 code)	n	%
122.0	*Echinococcus granulosus* infection of liver	1,411	9.9
122.1	*Echinococcus granulosus* infection of lung	467	3.3
122.2	*Echinococcus granulosus* infection of thyroid	3	0.0
122.3	*Echinococcus granulosus* infection, other	227	1.6
122.4	*Echinococcus granulosus* infection, unspecified	14	0.1
122.5–122.7	*Echinococcus multilocularis* infections	249	1.8
122.8	Echinococcosis, unspecified, of liver	9,632	67.6
122.9	Echinococcosis, other and unspecified	2,256	15.8
**Total**		**14,259**	**100.0**

For each entry, we collected socio-demographic (sex, age and autonomous community of residency) and clinical data (type and department of admission, length of hospitalization, non-invasive procedures and history of surgical intervention during the hospitalization, re-admission, outcome, hospitalization′s cost to the health care system, financing regime and diagnosis related group (DRG)). The length of hospitalization was computed by using the admission and the discharge dates for each hospitalization record. Costs were calculated using diagnostic cost group. The diagnostic cost group is based on the DRG for the hospitalized patients, age, sex, and resource consumption. DRG represents a medical-economic entity concerning a set of diseases requiring analogous management resources.

We described the clinical characteristics for all hospitalizations with echinococcosis as first diagnosis, and separately for: a) CE with liver infection; b) CE with lung infection; and c) unspecified CE hospitalizations and/or with other organs infestation. Differences in proportions between these groups were assessed by the χ2 test and the Student's t test for qualitative and quantitative data, respectively. We used two-sided tests and p < 0.05 was considered significant.

The average number of hospitalizations per year and the annual hospital admissions rate (per 100,000 per year) were calculated. Population at risk was obtained from the Spanish census projection [20]. It was assumed that the age distribution of the population covered by these hospitals was similar to the general population. Hospitalization rates were computed by autonomous community and year in order to assess temporal and geographical patterns. Trends in hospitalization rates were analyzed by linear regression for the whole study population and by age groups. Results in terms of mean rates by CC.AA were plotted in maps for the whole study period. The changes in rates over time were mapped using the Geographical Information System Arcgis version 10.0.

Data analysis was performed using STATA software version 12.

### Ethics statement

This study involves the use of patient medical data from The Spanish National Hospital Database (CMBD). These data are hosted by the Ministry of Health Social Services and Equality (MSSSI). Researchers working in public and private institutions can request the databases by filling, signing and sending a questionnaire available at the MSSSI website. In this questionnaire a signed Confidentiality Commitment is required. All data are anonymized and de-identified by the MSSSI before it is provided to applicants. According to this Confidentiality Commitment signed with the MSSSI, researchers cannot provide the data to other researchers that must request the data directly to the MSSSI [17].

## Results

A total of 14,259 hospital discharges with diagnosis of echinococcosis in first diagnostic position were identified for the 16-year study period. *E*. *granulosus* or non-specified echinococcosis (ICD-9-CM codes: 122.0, 122.1, 122.2, 122.3, 122.4, 122.8, and 122.9) were registered as etiological agent in 14,010/14,259 (98.3%) hospitalizations whereas *E*. *multilocularis* (ICD-9-CM codes: 122.5–122.7) was registered as etiological agent in 249/14,259 (1.7%) hospitalizations.

Excluding *E*. *multilocularis* records, organ involvement in the other 14,010 hospitalizations was encoded as follow: -Infection of the liver in 11,043 (78.8%); -Infection of the lung in 467 (3.3%); -Infection of other organs in 230 (1.6%); and; -Unspecified site in 2,270 (16.2%) (Table 1).

### Clinical characteristics of cystic echinococcosis in Spain

The mean age of the 14,010 hospitalization records was 55 years (range 0–99) with the 46–64 age group being slightly more represented (34.8%). Of the total 14,010 hospitalizations, 8,411 (60%) had registered a surgical intervention. The predominant admission type was “programmed” (60.6%). The 95.2% of hospitalizations were discharged at home, death occurring in 1.7%.

Overall, 1,673/14,010 (11.9%) were re-admissions. The hospitalization median time was 11 days and the hospitalization median cost ranged from 1,320.4 to 5,865,329 €; being the median cost per hospitalization of 8,439.1 €.

*Echinococcus granulosus* infection of the lung (ICD-9: 122.1) and unspecified echinococcosis (ICD-9: 122.3–4 and 122.9) were more common among males (p<0.005) while no significant sex differences were observed for CE hospital discharges with liver infection. Regarding the age distribution, the 11.35% of CE hospitalizations with lung infection were children under 15 years old. Compared to this rate, the percentage of children with liver infection or other organs/unspecified type were lower (1.85% and 4.52%, respectively). Surgical intervention occurred more commonly in CE hospital discharges with liver infection than in CE hospitalizations with lung infection (p<0.005). The 1.67% of CE hospital records with liver infection were exitus, percentage slightly lower than in CE records with lung infection (0.64%).

### Spatial and temporal trends in Spain

**Fig 1:**
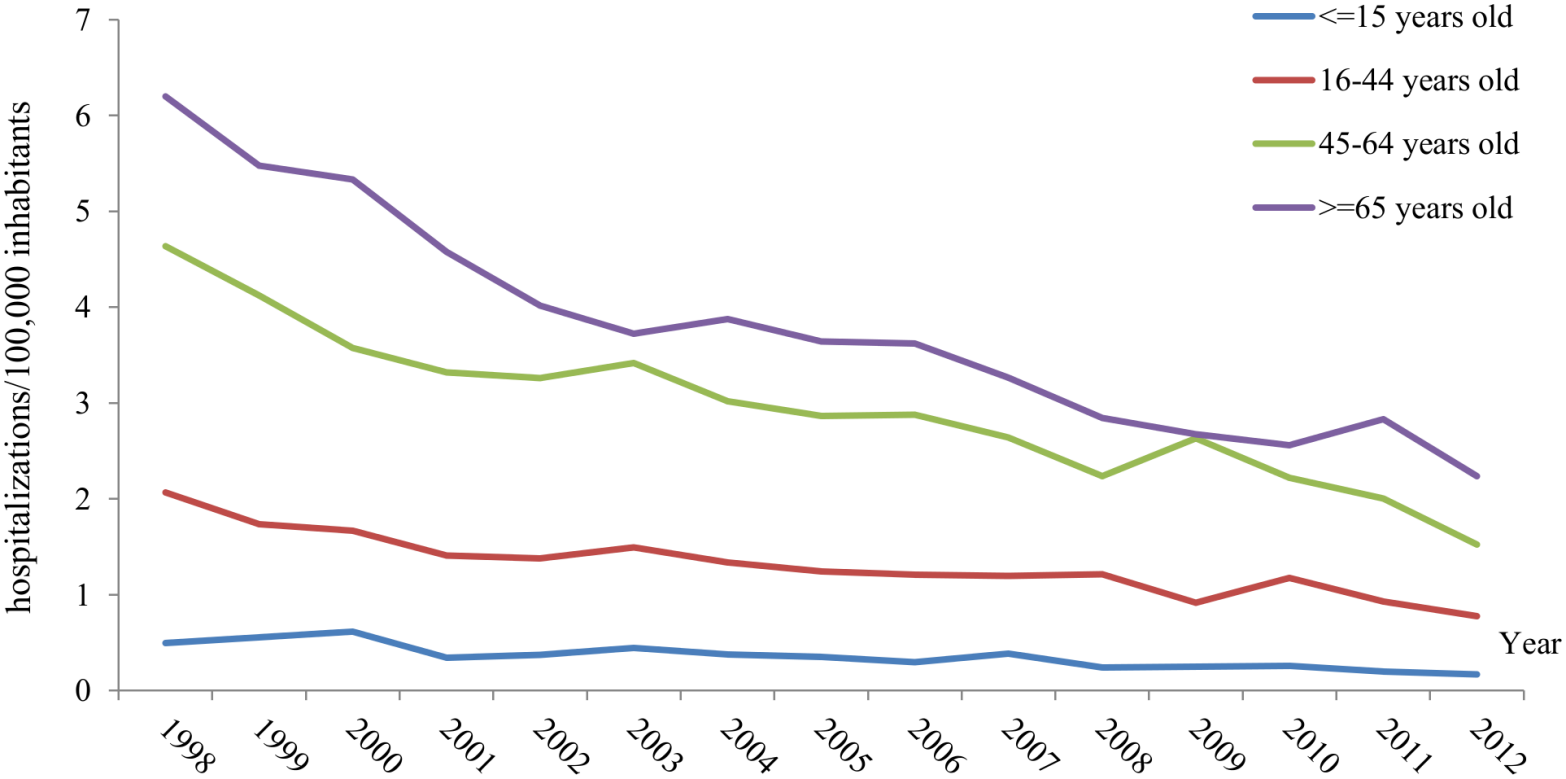
Mean rates (hospitalizations/100,000 per year) of cystic echinococcosis hospitalizations by age group and year, 1998–2012, Spain.

The temporal distribution of CE hospitalizations during the 16-year study period is represented in Fig 1. At the national level, the overall mean of yearly hospital discharges with a diagnosis of CE was 2.1/100,000 per year (range 1.9–2.2/100,000 per year). From 1997 to 2012, a decreasing trend was found (p<0.005) (S1 Table).

The 58.2% (8,160/14,010) of these hospitalizations were recorded between 1997 and 2004. For the two study periods (1998–2004 and 2005–2012), we obtained hospitalization rates of 2.5 and 1.6/100,000 per year, respectively. Hospitalization rates declined in all age groups during the study period. Pediatric CE hospitalizations were reported every year during the study period (n = 370, Table 2). Significant declines in hospitalization rates existed for all the age groups, although it was more accentuate for hospitalized patients aged 45–64 years old and those up to 64 years old (p<0.001) (Fig 1 and S2 Table).

**Table 2:** Clinical characteristics of cystic echinococcosis hospitalizations, 1997–2012, Spain.

Variable		Total	liver infection	Lung infection	Other organs/unspecified
		(n = 14,010)	(n = 11,043)	(n = 467)	(n = 2,500)
		N (%)	N (%)	N (%)	N (%)
**Sex**	**Male**	7,693 (54.92)	5,801 (52.54)	315 (67.45)	1,577 (63.08)
	**Female**	6,315 (45.08)	5,240 (47.46)	152 (32.55)	923 (36.92)
**Age-groups**	**<15 y**	370 (2.64)	204 (1.85)	53 (11.35)	113 (4.52)
	**16–44 y**	4,231 (30.20)	3,331 (30.17)	171 (36.62)	729 (29.16)
	**45–64 y**	4,882 (34.85)	3,876 (35.10)	132 (28.26)	874 (34.96)
	**> = 65 y**	4,526 (32.31)	3,631 (32.88)	111 (23.77)	784 (31.36)
**Surgical intervention**	**No**	5,599 (39.96)	4,248 (38.47)	237 (50.75)	1,114 (44.56)
	**Yes**	8,411 (60.04)	6,795 (61.53)	230 (49.25)	1,386 (55.44)
Type of admission*	**Urgent**	5,478 (39.10)	4,234 (38.34)	214 (45.82)	1,030 (41.20)
	**Programmed**	8,497 (60.65)	6,783 (61.42)	251 (53.75)	1,463 (58.52)
	**Others/unknown**	35 (0.25)	26 (0.24)	2 (0.43)	7 (0.28)
**Type of discharge**	**Home**	13,341 (95.22)	10,567 (95.69)	442 (94.65)	2,332 (93.28)
	**Transfer**	307 (2.19)	196 (1.77)	19 (4.07)	92 (3.68)
	**Others/unknown**	130 (0.93)	96 (0.87)	3 (0.64)	31 (1.24)
	**Exitus**	232 (1.66)	184 (1.67)	3 (0.64)	45 (1.80)
**Readmission**	**No**	12,337 (88.06)	9,742 (88.22)	402 (86.08)	2,193 (87.72)
	**Yes**	1,673 (11.94)	1,301 (11.78)	65 (13.92)	307 (12.28)
		**median (range)**	**median (range)**	**median (range)**	**median (range)**
**Hospitalization time (days)**		11	11	10	10
		(0–442)	(0–251)	(0–113)	(0–442)
**Hospitalization cost (euro)**		8,439.11	8,793.55	7,135.9	6,520.48
		(1320.36–5,865,329)	(1,320.36–5,865,329)	(2,062.73–1,554,050)	(2,433.08–5,096,296.50)

* “Urgent”: the patient required immediate attention for the care and treatment of his/her disease. The patient is admitted to the first available and suitable accommodation. “Programmed” (or elective): the patient's condition permitted adequate time to schedule the availability of suitable accommodations. “Other”: non-specified in the CMBD database

**Fig 2:**
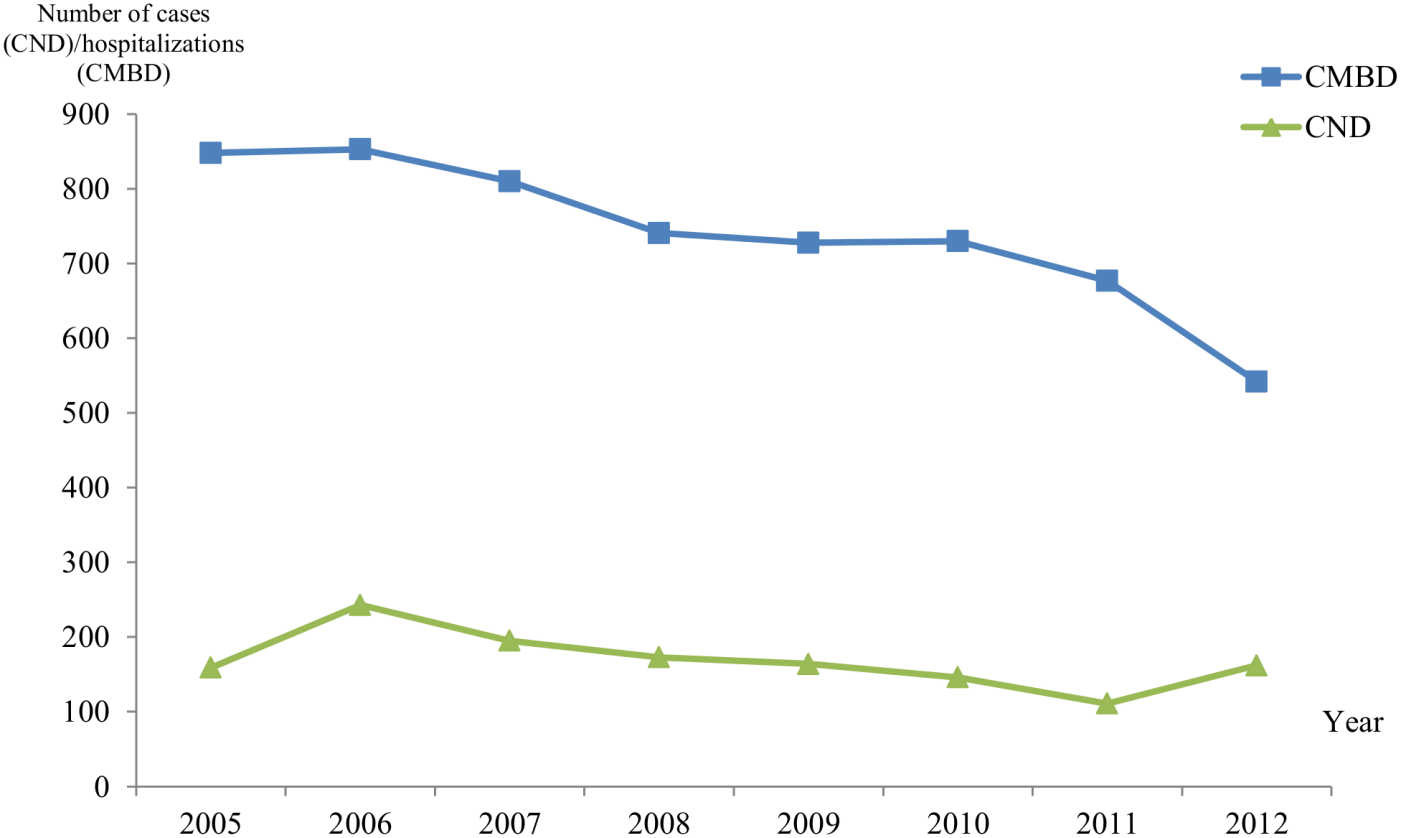
Comparison of the number of hospital registries (CMBD) of CE patients (blue line) with those reported in the CNDs (green line), from 2005 to 2012.

Comparison of the CE hospital registries and the data officially reported in the CND [21] is shown in Fig 2 (from 2005 to 2012). The figure shows that the number of CE cases reported in CND is much lower every year than the number of CE hospital discharges.

Regarding the regional distribution of CE hospitalizations as first diagnosis throughout the whole study period, CE hospitalizations occurred in all the CC.AA. Extremadura had the highest mean hospitalization rate (6.8 hospitalizations/100,000 per year), followed by Castilla-Leon (5.3/100,000 per year), Aragon (5.1/100,000 per year) and Castilla-La Mancha (4.4/100,000 per year). The insular CC.AAs of Spain (Balearic and Canary islands) were among the ones with lower CE hospitalization rates (S3 Table).

**Fig 3:**
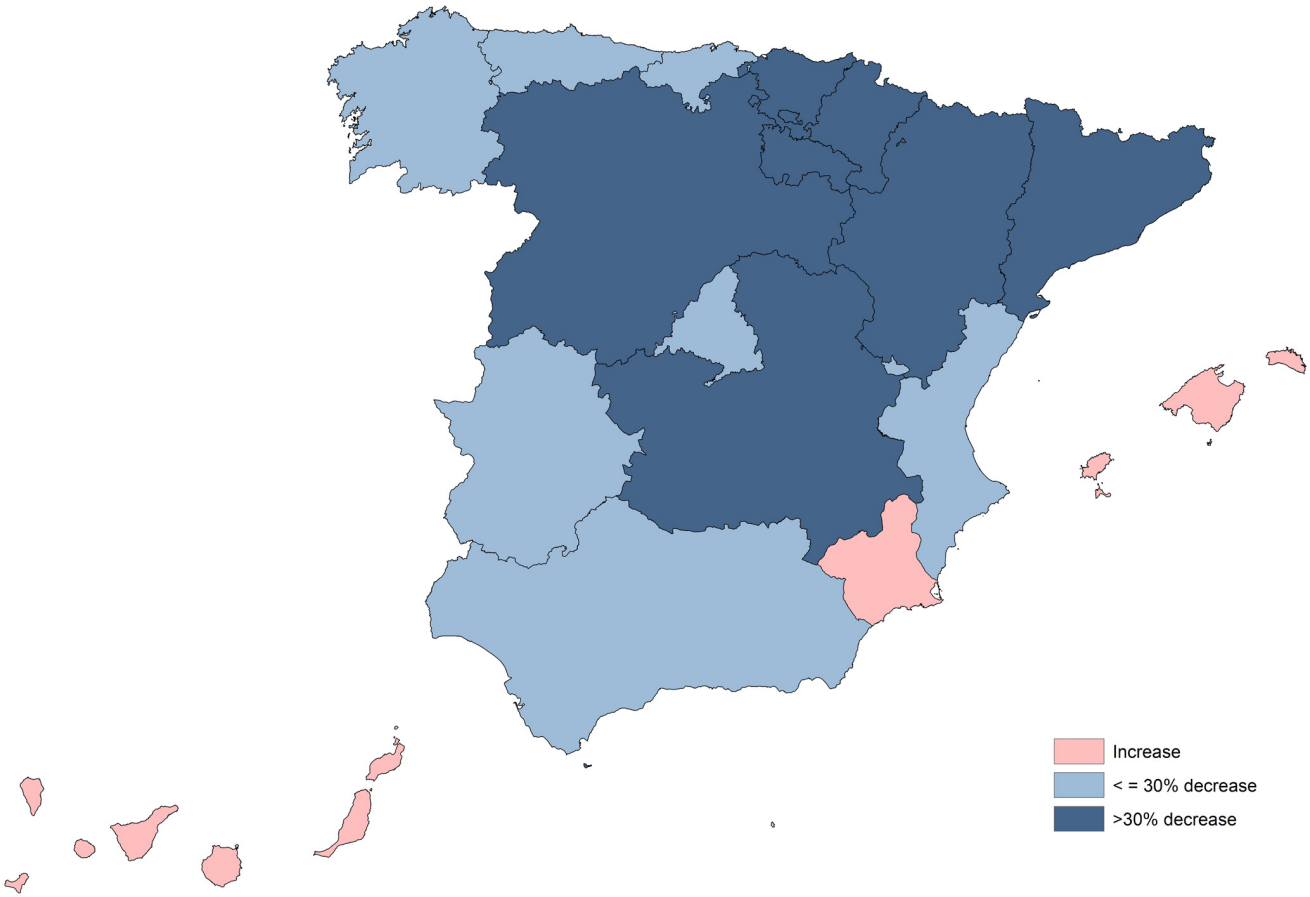
Comparative evolution of human echinococcosis hospitalizations rates (hospitalizations/100,000 per year) between 1997 and 2012, by autonomous community and time period, Spain.

For both study periods (1998–2004 and 2005–2012), the highest rates were recorded in the same autonomous communities (Extremadura, Castilla-Leon and Aragon). A significant decrease in the hospitalization rates was registered in all but three autonomous communities (Balearic and Canary Islands and Murcia) and one autonomous city (Melilla); these communities and city being among the ones with the lowest rates in the first period. The biggest decreases (≥50%) occurred in Ceuta and Navarra, followed by Rioja, Catalonia, Castilla-Leon and Basque Country, with up to 40% of decrease (Fig 3 and S3 Table).

**Fig 4:**
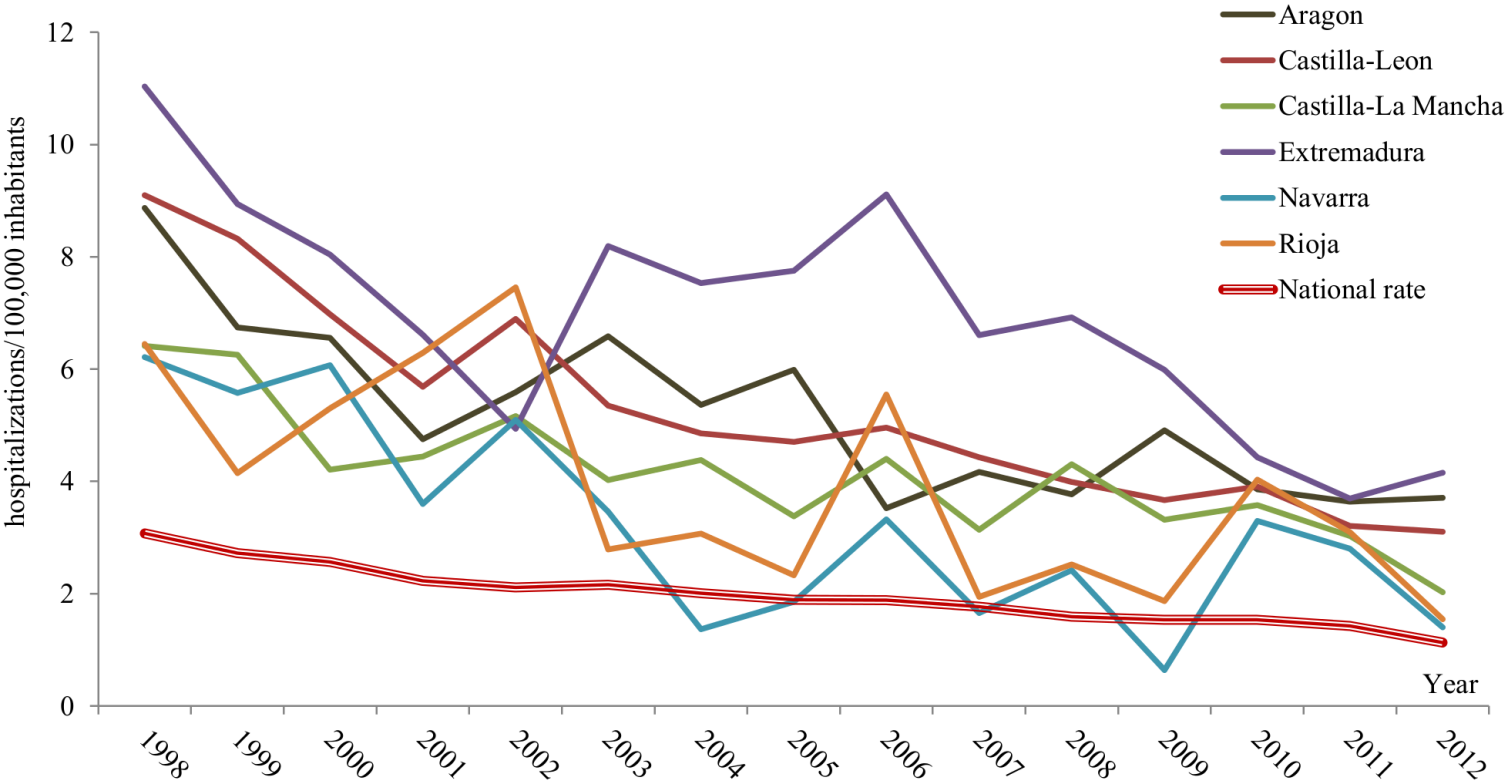
Mean rates (hospitalizations/100,000 per year) of cystic echinococcosis hospitalizations by autonomous community and year, in those communities with the highest rates, 1998–2012, Spain.

The hospitalization trends in those CC.AAs with higher rates revealed different patterns. In Extremadura, CE mean rates decreased from 1998 to 2002 and then peaked over national mean rates in 2003 and 2006, followed by a steady decline (except for 2012). In la Rioja and Castilla-Leon, similar peaks were registered in 2002 and 2006, both exceeding the national rate. In other communities, the hospitalization rate periodically reached high values during 2002, 2006, 2008 and 2010 (Fig 4).

## Discussion

CE is an endemic disease in Spain, although its burden and costs are difficult to estimate. Despite being subject to surveillance according to European legislation [22], notification of human cases ceased to be compulsory in 1995 [12]. Currently, health authorities are required to provide only a yearly summary of regional data [14]. In this sense, the deficiency of reporting systems of CE results in under-reporting and misreporting of this disease (13). This in turn creates the wrong opinion that CE is not an important health problem, leading to difficulties in the appropriate measurement of disease burden and impact on public health of CE.

Although there are some studies performed locally in Spain (13,15), to our knowledge, this is the first one addressing CE epidemiology nationwide. The comparison of officially notified cases (CND) with hospital records performed here indicates a clear discrepancy between both registries, with consistent underestimation of the burden of CE in official data, as already stated by other authors for some autonomous communities [8,15]. Nevertheless, this comparison should be read with caution; the number of CE cases might be overrated due to re-admissions while the CND data only gather information from some CC.AA. In Europe, difficulties in reporting of CE do not apply to Spain alone [23]. Comparison of notified cases with hospital records have indicated a clear discrepancy in several countries, with consistent underestimation of the burden of CE [8,23,24].

During the study period of 16 years, there were 14,010 hospitalizations for which *E*. *granulosus* or non-specified echinococcosis were recorded in first diagnostic position. The majority were recorded as non-specified hepatic echinococcosis. Surprisingly, we found 249 hospitalizations which included *E*. *multilocularis*. We decided to exclude this data from our analysis. In Spain, AE is a very rare disease; only two isolated cases have been reported in the literature [25,26]. Moreover, there are no data demonstrating the presence of the infection in the definitive hosts, neither exist standardized epidemiological studies showing the opposite [27]. The relatively high number of *E*. *multilocularis* hospitalizations that we found, might be most probably due to recording errors, considering that “multilocularis” may also be interpreted as “several locations” in Spanish. On the other hand, these AE related hospitalizations might correspond to imported cases. Unfortunately we lack information in the CMBD database to discard or support these explanations. In this sense, changes in CE codes are expected within the coming implementation of the ICD-10 in all Spanish hospitals in January 2016 [28]. In our opinion, this new CE classification will fit better the clinical spectrum of this disease. Therefore, more accurate recording process might be expected in the future.

Besides inaccurate reporting of the number of cases, there are additional limitations in the current reporting systems of CE, e.g., the lack of discrimination between autochthonous and imported cases. In this sense, Lopez-Bernus et al. [15] have recently reported that immigrants represent only the 3% of CE hospital admissions in public hospitals from Castilla-Leon. However, results from Castilla-Leon may not be representative for the rest of the country, as this is one of the Spanish CC.AA with lower percentage of immigrant population [20]. Further limitations on the current report of CE with impact in the management of patients and in the estimation of the epidemiological situation of CE have been recently revised in Italy by Tamarozzi et al.[24].

Our study indicates that most of the CE hospitalizations were males aged over 16 years old. In a research carried out in the United States, the age-adjusted mortality rates were also higher in males than in females [29]. This apparent increased risk of echinococcosis in males may reflect higher rates of occupational exposures from the tending of livestock. The even distribution of hospitalized cases in persons older than 16 supports this finding. Moreover, one third of the hospitalizations occur in each of the age groups 16–44, 45–64 and > 65 years old, which might suggest that CE infection occurs in youth ages. On the other hand, hospitalizations in children persisted over the years despite of the control measures. Children can be more susceptible to get infected through fecal–oral contact with dogs, particularly in the course of playful and intimate contact between children and dogs [30]. Even the decline over the study period was less pronounced for this age group. This might indicates an active transmission of disease and a lack of success of the regional control and prevention programs for CE, thus much remains to be done.

Overall, liver was the most frequent reported site of infection, which is expected, as liver involvement is the most common manifestation of echinococcosis [3]. We found a ratio lung:liver involvement higher in children than in adults, which has been previously described in the literature. In adult humans, cysts occur more frequently in the liver, followed by the lungs [31]. In children, lung is the predominating site for cyst detection apparently by allowing faster growth of the cyst due to its compressible nature, vascularization, and negative pressure [32].

Around 60% of the overall CE hospitalizations were submitted to surgery. In the past, surgery was the only treatment for cystic echinococcal cysts. Currently, four options exist for the clinical management of CE: percutaneous treatment of the hydatid cysts with the PAIR (Puncture, Aspiration, Injection, Re-aspiration) technique, surgery, anti-infective drug treatment, and “watch and wait” [33]. In this study, 60% of hospital discharges with liver localization went under surgery, which is a bit striking knowing that other several non-surgical techniques (such as PAIR) exist for this clinical manifestation. On the other hand, only 50% of CE patients with lung infection were operated, despite that surgery is the main therapeutic approach in pulmonary cystic echinococcosis [32]. Therefore, in our study pulmonary CE got less surgery than liver CE. We know that the frequency of surgical interventions is usually higher in pulmonary hydatid location [34], and that the most commonly affected organ in CE is the liver, followed by the lung [3]. In our database, only 3.3% of CE hospitalizations were encoded as pulmonary location. Pulmonary involvement usually coexists (up to 75%) with concomitant liver involvement in adult patients [35]. Thus, a possible explanation could be that some hospitalizations were encoded as liver hydatid disease, although lung involvement existed too. Moreover, hepatopulmonary hydatidosis might have also been wrongly recorded as multilocularis echinococcosis or "other / unspecified". Unfortunately, we cannot disentangle this issue with the CMBD database.

The median cost per hospitalization was 8,439.11 €, ranging from 1,320.4 to 5,865,329 euros. Compare to the public health care expenses per year in Spain (median cost per inhabitant of 1,510 € for the year 2009, [36]) and the hospitalization costs for cardiovascular diseases, one of the most prevalent diseases in Spain, for the same year (median 5,732.9 € per patient [37]), the hospitalization median cost per CE patient was much higher. Echinococcosis is often expensive and complicated to treat, and may require extensive surgery and/or prolonged drug therapy [38], causing more expenses per patient than the expenses related with hospitalization of cardiovascular diseases patients. Thus, the need for effective disease control measures is also key from an economic point of view.

Hospitalization time and cost were lower in patients with lung involvement than liver infection, as well as exitus rates, which could means that pulmonary form is less severe. Cysts in the lung are not usually life-threatening. Rather, it is the underlying cyst in the liver that is responsible of major CE complications. Besides, cysts in the liver resist treatment and are often not fully resectable, whereas pulmonary cysts use to respond well to standard and are more easily surgically removed [39].

The overall rate of yearly hospital discharges with CE diagnosis in the present study was 2.1; with a significant decreasing trend from 1998–2004 to 2005–2012. In Spain, the implementation of control programs for echinococcosis started in the 1980s, mainly based on the periodic treatment of dogs with praziquantel [8], and it led to a significant decrease in human incidence rates according to the CND reports; in 1985, there were about 1,000 new human cases (about 2.5/100,000 per year) and the incidence rate dropped to 0.78/100,000 per year in 1997 [10]. To our knowledge, the rate of CE has not been updated since then, coinciding with the implementation of the new decentralized surveillance system in 1996 [13].

In our study, the geographical distribution of CE hospitalizations, as well as the rate change between the two study periods, varied among CC.AA. Extremadura had the highest mean hospitalization rate for the whole period, followed by Castilla-Leon, Aragon and Castilla-La Mancha, while major rates ‘decrease occurred in Navarra and Ceuta. In other autonomous communities the decrease was less substantial (i.e. Galicia), or even opposed (three autonomous communities and one autonomous city, Balearic Islands, Canary Islands, Murcia and Melilla, shown rates´ increase between both study periods). As expected, the endemic CC.AA with higher rates had experienced a decline (probably due to the control measures), while CE hospitalization rates increased in the CC.AA where the presence of the disease has been rarely (Balearic Islands [40,41],or very rarely described (in Canary Islands, the first autochthonous case was reported in 1985, not existing evidence of any new case since then [42]). Nevertheless, it should be noticed that the place of origin of the infection is not reported in the CMBD database.

Regarding the regional trends, we observed peaks in 2002, 2006, 2008 and 2010 for most CC.AAs with higher CE incidence, although differences also exist among them. Spatial and temporal clusters of CE have been previously described in France and Switzerland [43]. The trend differences that we found may be explained by disparities in the regional health programs after 1995, when the CC.AA became fully in charge of the financing and implementation of the control programs [44]. Other possible explanation is the migratory movement, both immigration and rural to urban migration within the country. Since CE is a chronic disease and the development of signs and symptoms lasts years, prevention and control programs as well as changes in population movement, could have an overdue impact, difficult to measure through an indicator such as the hospital discharge diagnoses [33]. Besides, the lack of reliable statistics on the epidemiology of echinococcosis in dogs and farm animals makes it difficult to compare prevalence rates before and after the application of control programs in Spain [8]. Whatever the case may be, our results underline the need of a nationwide systematic approach towards CE.

### Limitations and conclusions

Even if the CMBD provide information from a network of hospitals that covers more than 99% of the population living in Spain [17,18], hospital discharge records do not include cases managed in an outpatient setting or asymptomatic cases, thus hospital records are still underestimating the real burden of CE in Spain. Moreover, we decided to select only those records with CE as main diagnosis, assuming that the first diagnostic position is the main cause of hospitalization. Otherwise, encoding error may exist, and cannot be amended as the data included in the CMBD are irreversible.

The CMBD does not provide information about parasite isolation, detection cyst stages, PCR or serologic test for diagnosis of echinococcosis, and this impairs the quality of data. However, the CMBD database provides reliable information to support decision-making based on ICD-9 codification carried out by medical doctors without being subject to the limitations of outpatient surveillance systems, such as under-diagnosis or reporting deficiencies. It remains dependent only by the population’s health seeking behavior and healthcare accessibility [45]. CMBD has also been proved to be a trusted source for many epidemiological studies [46].

Important additional information to assess CE epidemiology such as occupational exposures and geographical origin is not collected in the CMBD database. Moreover, we also lack of information from the animal side at national level. Data about disease prevalence in dogs in Spain are only found in a few publications, and are frequently biased since there is no consensus on what should be measured or the diagnostic method to use [47]. Data from farm animal echinococcosis prevalence reported to the EFSA are obtained on slaughtered animals by inspection each year. Even so, these are probably underestimations, as the vast majority of slaughtered animals are less than 1 year old, less than the time needed for the oncosphere to develop into a visible lesion [8].

In summary, available information on CE is incomplete and insufficient to assess properly its epidemiology in Spain, despite that CE remains one of the most important anthropozoonoses in terms of human incidence and morbidity [9]. A comparison of notified cases with data from hospital records has previously shown that CE case numbers have been clearly underestimated in the last 10 years [11]. Furthermore, Benner et al. calculate the overall economic losses due to human and animal CE in Spain in 2005; assuming no underreporting, they estimated an annual loss of €148,964,534 [48]. All these figures underline the need of a national surveillance system, which would allow a more accurate data collection, analyzing and interpretation. It will also be of added value in completing a more accurate picture of CE in Spain, resulting useful both in gaining extended disease knowledge and reducing CE morbidity and related-costs, but especially in evaluating implemented control actions.

Due to the limitations of current reporting systems, the WHO Collaborating Centre for the Clinical Management of Cystic Echinococcosis, (University of Pavia, San Matteo Hospital Foundation, in Pavia) and the Istituto Superiore di Sanità (the Italian National Health Institute, ISS, in Rome) set up the Italian registry of cystic echinococcosis (Registro Italiano Echinococcosi Cistica, RIEC) as an initiative at national level in Italy [24]. This has been recently scaled up to the European registry for cystic echinococcosis (http://www.heracles-fp7.eu/erce.html,), an online registry that allows the collection of both basic epidemiology and detailed longitudinal clinical data in a simple and unequivocal way, in an initiative funded by the European Union [49]. This tool provides an efficient and disease-tailored template to governments, the European Commission and related European agencies to harmonise data collection, monitoring and reporting of CE. The planned publication of the first data of this registry in the near future could help to foster the participation of clinicians managing CE patients at European level, thus giving the chance to evaluate more accurately the burden of disease cause by CE in the European Union.

## Supporting Information

S1 Table Cystic echinococcosis hospitalizations rates per 100,000 per year, 1997–2012, Spain. (DOCX)

S2 Table Cystic echinococcosis hospitalizations rates per 100,000 per year by age group, 1997–2012, Spain. (DOCX)

S3 Table Cystic echinococcosis hospitalizations rates per 100,000 per year by autonomous community and time period, 1997–2012, Spain. (DOCX)

S1 Checklist STROBE Checklist. (DOC)
