# Supplementary material for: Cystic Echinococcosis Epidemiology in Spain Based on Hospitalization Records, 1997-2012
Source: PLoS Negl Trop Dis. 2016 Aug 22;10(8):e0004942. doi: 10.1371/journal.pntd.0004942 (PMC4993502; doi:10.1371/journal.pntd.0004942)
Supplement: S1 Table — (DOCX) [file pntd.0004942.s001.docx]

| **Supplementary table 1. Cystic echinococcosis hospitalizations rates per 100,000 per year, 1997-2012, Spain.** | | | |
| --- | --- | --- | --- |
| **Year** | **Population** | **Total** | |
|  |  | **Cases** | **Rate*100,000** |
| **1997** | 39,761,022 | 1,216 | 3.06 |
| **1998** | 39,852,651 | 1,224 | 3.07 |
| **1999** | 40,202,160 | 1,093 | 2.72 |
| **2000** | 40,499,791 | 1,037 | 2.56 |
| **2001** | 41,116,842 | 916 | 2.23 |
| **2002** | 41,837,894 | 882 | 2.11 |
| **2003** | 42,717,064 | 922 | 2.16 |
| **2004** | 43,197,684 | 870 | 2.01 |
| **2005** | 44,108,530 | 835 | 1.89 |
| **2006** | 44,708,964 | 839 | 1.88 |
| **2007** | 45,200,737 | 801 | 1.77 |
| **2008** | 46,157,822 | 734 | 1.59 |
| **2009** | 46,745,807 | 716 | 1.53 |
| **2010** | 47,021,031 | 721 | 1.53 |
| **2011** | 47,190,493 | 669 | 1.42 |
| **2012** | 47,265,321 | 535 | 1.13 |
