# Supplementary material for: Cystic Echinococcosis Epidemiology in Spain Based on Hospitalization Records, 1997-2012
Source: PLoS Negl Trop Dis. 2016 Aug 22;10(8):e0004942. doi: 10.1371/journal.pntd.0004942 (PMC4993502; doi:10.1371/journal.pntd.0004942)
Supplement: S2 Table — (DOCX) [file pntd.0004942.s002.docx]

| **Supplementary table 2. Cystic echinococcosis hospitalizations rates per 100,000 per year by age group, 1997-2012, Spain.** | | | | |
| --- | --- | --- | --- | --- |
| **Year** | **Age group** | | | |
|  | **<=15 years old** | **16-44 years old** | **45-64 years old** | **>=65 years old** |
| **1998** | 0.50 | 2.07 | 4.64 | 6.20 |
| **1999** | 0.56 | 1.73 | 4.12 | 5.48 |
| **2000** | 0.61 | 1.67 | 3.58 | 5.33 |
| **2001** | 0.34 | 1.41 | 3.32 | 4.58 |
| **2002** | 0.37 | 1.38 | 3.26 | 4.02 |
| **2003** | 0.45 | 1.50 | 3.42 | 3.72 |
| **2004** | 0.38 | 1.34 | 3.02 | 3.88 |
| **2005** | 0.35 | 1.24 | 2.87 | 3.64 |
| **2006** | 0.30 | 1.21 | 2.88 | 3.62 |
| **2007** | 0.39 | 1.20 | 2.64 | 3.27 |
| **2008** | 0.24 | 1.21 | 2.24 | 2.84 |
| **2009** | 0.25 | 0.92 | 2.63 | 2.67 |
| **2010** | 0.26 | 1.17 | 2.22 | 2.56 |
| **2011** | 0.20 | 0.93 | 2.00 | 2.83 |
| **2012** | 0.17 | 0.78 | 1.52 | 2.24 |
