# Supplementary material for: Cystic Echinococcosis Epidemiology in Spain Based on Hospitalization Records, 1997-2012
Source: PLoS Negl Trop Dis. 2016 Aug 22;10(8):e0004942. doi: 10.1371/journal.pntd.0004942 (PMC4993502; doi:10.1371/journal.pntd.0004942)
Supplement: S3 Table — (DOCX) [file pntd.0004942.s003.docx]

| **Supplementary Table 3. Cystic echinococcosis hospitalizations rates per 100,000 per year by autonomous community and time period, 1997-2012, Spain.** | | | | | |
| --- | --- | --- | --- | --- | --- |
| **Autonomous community** | **Total 1998-2012** | **First period 1998-2004** | **Second period 2005-2012** | **Hospitalization rate changes (times)** | **Change direction** |
| **Andalusia** | 0.92 | 1.07 | 0.78 | 0.73 | **↓** |
| **Aragon** | 5.08 | 6.09 | 4.20 | 0.69 | **↓↓** |
| **Asturias** | 0.61 | 0.73 | 0.51 | 0.70 | **↓** |
| **Balearic Islands** | 0.61 | 0.48 | 0.73 | 1.52 | **↑** |
| **Canary Islands** | 0.16 | 0.12 | 0.19 | 1.60 | **↑** |
| **Cantabria** | 0.93 | 1.04 | 0.84 | 0.81 | **↓** |
| **Castilla-Leon** | 5.27 | 6.73 | 3.99 | 0.59 | **↓↓** |
| **Castilla-La Mancha** | 4.40 | 5.32 | 3.59 | 0.67 | **↓↓** |
| **Catalonia** | 1.14 | 1.50 | 0.84 | 0.56 | **↓↓** |
| **Valencia** | 1.51 | 1.77 | 1.29 | 0.73 | **↓** |
| **Extremadura** | 6.79 | 7.83 | 5.88 | 0.75 | **↓** |
| **Galicia** | 0.86 | 0.91 | 0.81 | 0.90 | **↓** |
| **Madrid** | 2.28 | 2.60 | 1.99 | 0.77 | **↓** |
| **Murcia** | 0.89 | 0.72 | 1.04 | 1.45 | **↑** |
| **Navarra** | 3.10 | 4.22 | 2.13 | 0.50 | **↓↓** |
| **Basque Country** | 1.94 | 2.49 | 1.46 | 0.59 | **↓↓** |
| **Rioja** | 3.81 | 5.07 | 2.70 | 0.53 | **↓↓** |
| **Ceuta** | 2.31 | 3.63 | 1.15 | 0.32 | **↓↓** |
| **Melilla** | 0.75 | 0.64 | 0.86 | 1.35 | **↑** |
| **↑** increase; **↓** ≤ 30% decrease; **↓↓** ≥30% decrease | | | | | |
